# Effect of cadence on locomotor–respiratory coupling during upper-body exercise

**DOI:** 10.1007/s00421-016-3517-5

**Published:** 2016-12-28

**Authors:** Nicholas B. Tiller, Mike J. Price, Ian G. Campbell, Lee M. Romer

**Affiliations:** 1 Academy of Sport and Physical Activity, Sheffield Hallam University, Sheffield, UK; 2 Division of Sport, Health and Exercise Sciences, Brunel University London, London, UK; 3 Faculty of Health and Life Sciences, Coventry University, Coventry, UK; 4 School of Life and Medical Sciences, University of Hertfordshire, Hatfield, UK

**Keywords:** Arm-cranking, Cardiorespiratory, Diaphragm, Entrainment, Respiratory muscle

## Abstract

**Introduction:**

Asynchronous arm-cranking performed at high cadences elicits greater cardiorespiratory responses compared to low cadences. This has been attributed to increased postural demand and locomotor–respiratory coupling (LRC), and yet, this has not been empirically tested. This study aimed to assess the effects of cadence on cardiorespiratory responses and LRC during upper-body exercise.

**Methods:**

Eight recreationally-active men performed arm-cranking exercise at moderate and severe intensities that were separated by 10 min of rest. At each intensity, participants exercised for 4 min at each of three cadences (50, 70, and 90 rev min^−1^) in a random order, with 4 min rest-periods applied in-between cadences. Exercise measures included LRC via whole- and half-integer ratios, cardiorespiratory function, perceptions of effort (RPE and dyspnoea), and diaphragm EMG using an oesophageal catheter.

**Results:**

The prevalence of LRC during moderate exercise was highest at 70 vs. 50 rev min^−1^ (27 ± 10 vs. 13 ± 9%, *p* = 0.000) and during severe exercise at 90 vs. 50 rev min^−1^ (24 ± 7 vs. 18 ± 5%, *p* = 0.034), with a shorter inspiratory time and higher mean inspiratory flow (*p* < 0.05) at higher cadences. During moderate exercise, $\dot{V}{\text{O}}_{ 2}$ and *f*_C_ were higher at 90 rev min^−1^ (*p* < 0.05) relative to 70 and 50 rev min^−1^ ($\dot{V}{\text{O}}_{ 2}$ 1.19 ± 0.25 vs. 1.05 ± 0.21 vs. 0.97 ± 0.24 L min^−1^; *f*_C_ 116 ± 11 vs. 101 ± 13 vs. 101 ± 12 b min^−1^), with concomitantly elevated dyspnoea. There were no discernible cadence-mediated effects on diaphragm EMG.

**Conclusion:**

Participants engage in LRC to a greater extent at moderate-high cadences which, in turn, increase respiratory airflow. Cadence rate should be carefully considered when designing aerobic training programmes involving the upper-limbs.

## Introduction

Asynchronous arm-cranking performed at moderate-high cadences (70–90 rev min^−1^) results in higher oxygen uptake ($\dot{V}{\text{O}}_{ 2}$) and minute ventilation ($\dot{V}_{\text{E}}$) at a given submaximal power output when compared to lower cadences (50–60 rev min^−1^) (Price et al. 2007). These observations might be due to several mechanisms. First, at a given power output, low cadence arm-cranking extends the stroke duty cycle relative to high-cadence arm-cranking and, thus, requires greater force output from the exerciser to overcome the increased flywheel inertia. This might, in turn, predispose the exerciser to an earlier onset of local neuromuscular fatigue during low cadence ergometry (Smith et al. 2001). Second, the greater force output associated with low cadences likely results in the recruitment of aerobically meagre fast-twitch (type II) fibres which retard the O_2_ response (Kushmerick et al. 1992). Third, the elevated $\dot{V}{\text{O}}_{ 2}$ at high cadences has been attributed to greater isometric contractions of the postural muscles to stabilise the torso, in addition to a greater prevalence of locomotor–respiratory coupling (Price et al. 2007).

Locomotor–respiratory coupling (LRC), also termed entrainment, refers to the phase-locking of locomotor and respiratory frequencies during exercise (Perseqol et al. 1991; O’Halloran et al. 2011). The principal mechanism underpinning LRC is yet to be elucidated, but is associated with peripheral neurogenic drive from moving limbs (Iwamoto et al. 2010), perhaps, conferring an energetic or perceptual advantage (Stickford et al. 2015). Earlier studies in cycling exercise observed that the coupling of breathing and cycling rhythms resulted in a reduced oxygen uptake at a given workload (Garlando et al. 1985). However, LRC has not widely been studied during upper-body exercise, and it is not known how LRC is influenced by the extensive thoracic muscle loading that characterises this activity. Indeed, since most respiratory muscles attach to the ribs or associated structures, these muscles function to ventilate the lungs while simultaneously stiffening the spine (Hodges et al. 2005) and maintaining torso stabilisation and arm position (Celli et al. 1988). Furthermore, the abdominal muscles contract dynamically to flex and rotate the torso, and the diaphragm aids postural stability prior to sudden, voluntary movements of the upper-limbs (Hodges et al. 1997). As such, upper-body exercise places substantial demand on muscles of the thorax for simultaneous postural, locomotor, and ventilatory tasks. Since contractions of trunk musculature are initiated to counteract disturbances to spinal stability caused by limb movement (Hodges and Gandevia 2000), it is likely that rapid arm movements exhibited during high-cadence arm-cranking will exacerbate the essential reactive postural demands of the thoracic muscles, particularly at high exercise intensities. This may further increase O_2_ consumption at high cadences. Because LRC may be initiated to facilitate airflow during periods of respiratory muscle antagonistic loading (Daley et al. 2013), it is possible that both postural loading and LRC would be exacerbated at high arm-crank cadences. As such, manipulating the arm-crank cadence provides an effective model with which to assess the relationship between cardiorespiratory function and LRC. Data to this effect will highlight the cadences that elicit optimal physiological responses and, therefore, might inform athletic training programmes that emphasise the upper-limbs. This data might also inform upper-body training or rehabilitation programmes for clinical populations.

Accordingly, the aim of the current study was to determine the acute effects of cadence and exercise intensity on cardiorespiratory function and prevalence of LRC during arm-cranking in healthy adults. We hypothesised that greater postural demand at high cadences would increase the reactive postural component, thereby increasing cardiorespiratory stress and necessitating an increased prevalence of LRC.

## Methods

### Participants

Eight healthy, non-smoking, recreationally-active men between the ages of 18 and 35 years volunteered to participate in the study (mean ± SD age 24 ± 4 years, stature 1.76 ± 0.05 m, mass 67.4 ± 6.4 kg, upper-body $\dot{V}{\text{O}}_{{ 2 {\text{peak}}}}$ 30.3 ± 4.5 mL kg^−1^ min^−1^). Experimental procedures were approved by the institution research ethics committee and were performed in accordance with the ethical standards as laid down in the 1964 Declaration of Helsinki (World Medical Association Declaration of Helsinki 2013), and participants provided written informed consent. Participants were asked to abstain from exercise for 48 h, alcohol and caffeine for 12 h, and food for 3 h prior to each visit.

### Experimental overview

All procedures were completed during three visits to the laboratory, each separated by a minimum of 2 days and no longer than 1 week. Exercise trials were conducted at the same time of day to eliminate any influence of circadian variance. At the first visit, participants completed anthropometry and pulmonary function tests, and were familiarised with arm-crank ergometry and the rating of perceptions. At the second visit, participants completed a maximal ramp incremental exercise test on an arm-crank ergometer for the determination of peak power output (*W*_peak_) and associated cardiorespiratory responses (e.g., $\dot{V}{\text{O}}_{{ 2 {\text{max}}}}$, gas-exchange threshold). At the third visit, participants performed moderate and severe constant-power arm-crank exercise at each of three cadences (50, 70 and 90 rev min^−1^), while LRC, diaphragm electromyography (EMG), cardiorespiratory function, and perceptual responses (RPE and ratings of dyspnoea) were assessed (see below).

### Visit 1, baseline pulmonary function

Participants were screened for pulmonary dysfunction using a fully integrated system with whole-body plethysmography (MasterScreen PFT Pro, CareFusion, Hampshire, UK). Airway resistance, slow and dynamic components of lung function, and diffusion capacity for carbon monoxide were assessed using recommended procedures (MacIntyre 2005; Miller et al. 2005; Wanger 2005). All participants were free from respiratory disorders (Table 1).

**Table 1:** Baseline pulmonary function

	Absolute	%Predicted
VC (L)	5.41 ± 0.92	105 ± 13
FEV_1_ (L)	4.36 ± 0.45	101 ± 9
FEV_1_/VC (%)	81.5 ± 6.8	89 ± 8
TLC (L)	7.3 ± 1.2	104 ± 11
RV (L)	1.93 ± 0.47	116 ± 26
FRC (L)	3.74 ± 0.96	114 ± 26
IC (L)	3.58 ± 0.62	95 ± 16
PEF (L s^−1^)	9.4 ± 1.5	94 ± 13
MVV (L min^−1^)	186 ± 20	108 ± 14
sRaw_eff_ (kPa s L^−1^)	0.77 ± 0.20	66 ± 17
Raw_eff_ (kPa^−1^)	0.19 ± 0.05	64 ± 18
*D*_L,CO_ (mmol min^−1^ kPa^−1^)	12.1 ± 1.3	102 ± 12
*V*_A_ (L)	7.13 ± 0.91	103 ± 8
*V*_I_ (L)	5.58 ± 0.68	103 ± 9

Mean ± SD, *n* = 8

*VC* vital capacity, *FEV*_*1*_ forced expiratory volume in one second, *TLC* total lung capacity, *RV* residual volume, *FRC* functional residual capacity, *IC* inspiratory capacity, *PEF* peak expiratory flow, *MVV* maximum voluntary ventilation in 12 s, *sRaw*_*eff*_ specific effective airway resistance, *Raw*_*eff*_ effective airway resistance, *D*_*L, CO*_ diffusion capacity for carbon monoxide (uncorrected for haemoglobin); *V*_*A*_ alveolar volume, *V*_*I*_ inspiratory volume

### Visit 2, maximal ramp test

Participants completed a maximal incremental exercise test on an electromagnetically braked arm-crank ergometer set in the hyperbolic mode (Angio, Lode, Groningen, The Netherlands). The ergometer was wall-mounted and positioned, so that the scapula-humeral joint and the distal end of the crank pedal were horizontally aligned. Participants were instructed to sit upright, maintain form at all times, and keep their feet flat to the floor to minimise bracing. After 3 min of rest, participants exercised for 3 min at 20 W after which the work rate was increased in a ramp fashion by 15 W min^−1^ with cadence standardised at 75 rev min^−1^ (Marais et al. 2002). The test was terminated when cadence dropped below 65 rev min^−1^ for more than 3 s despite verbal encouragement. Cardiorespiratory variables were assessed continuously (see below) and peak values recorded in the final 30 s of maximal exercise. Gas-exchange threshold was identified using multiple parallel methods (Wasserman 1984; Beaver et al. 1986).

### Visit 3, varied-cadence, constant-power test

The varied-cadence, constant-power test comprised 2 × 12 min bouts of arm-cranking, each bout separated by 10 min passive rest. Within each 12 min bout, participants exercised at 50, 70, and 90 rev min^−1^ in 4 min efforts with 4 min of passive rest between each cadence to minimise carry-over effects. The work rate for each 12 min bout was equivalent to 80% of gas-exchange threshold (moderate) and 65% of the difference between gas-exchange threshold and $\dot{V}{\text{O}}_{{ 2 {\text{peak}}}}$ (severe) (Lansley et al. 2011). The required work rate calculated for the constant-power test was reduced by two-thirds of the increment that was applied during the maximal ramp test (i.e., 10 W) to accommodate for the mean lag time in O_2_ uptake kinetics that has been observed during ramped exercise (Whipp et al. 1981). Participants exercised at moderate then severe work rates to minimise fatigue, whereas cadence order was randomised.

#### Locomotor–respiratory coupling

**Fig. 1:**
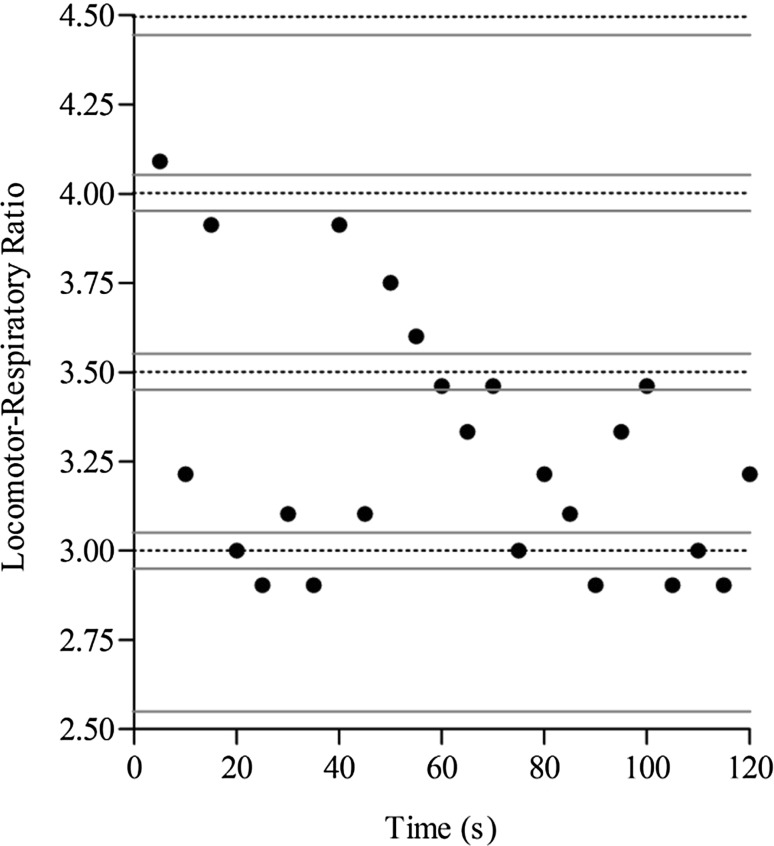
Locomotor–respiratory ratio calculated at 5 s intervals for a representative participant performing severe-intensity arm-crank exercise at 90 rev min^−1^. Locomotion and respiration were considered to be matched when the instantaneous ratio recorded at 5 s intervals was within ±0.05 of a whole- or half-integer value. The prevalence of LRC in this representative example was 25%

Locomotor and respiratory rhythms were considered to be matched when the instantaneous ratio of cadence (50, 70, or 90 rev min^−1^) to mean respiratory frequency, recorded at 5 s intervals, was within ±0.05 of a whole- or half-integer value (Paterson et al. 1986; Fig. 1). The prevalence of locomotor–respiratory coupling (%LRC) was calculated as the percentage of the sampled data within each bout of exercise that met these criteria.

#### Diaphragm electromyography

Neuromuscular activation of the crural diaphragm (EMG_di_) was assessed using a bespoke multi-pair esophageal electrode catheter (Gaeltec Devices Ltd., Dunvegan, Isle of Sky, UK). The catheter comprised a 100 cm silicon shaft (2.7 mm diameter) with 7 platinum electrodes spaced 1 cm apart. With the subject resting in the seated position, the catheter was passed pernasally into the stomach, re-positioned based on the strength of the EMG_di_ recorded simultaneously from different pairs of electrodes (Luo et al. 2008), and anchored in place with surgical tape. EMG_di_ was normalised against the highest root mean square (RMS) recorded during a maximal inspiratory manoeuvre performed from functional residual capacity.

#### Cardiorespiratory responses

Cardiorespiratory function was assessed via the continuous measurement of cardiac frequency (*f*_C_) by telemetry (Vantage NV; Polar Electro Oy), arterial oxygen saturation (SpO_2_) using forehead pulse oximetry (OxiMax N-560, Nellcor, Tyco Healthcare, Pleasanton, CA, USA), and breath-by-breath indices of pulmonary ventilation and gas exchange using online gas analysis (Oxycon Pro, Jaeger GmbH).

#### Perceptual responses

In the final 30 s of each 4 min effort, participants were asked to rate their “intensity of breathing discomfort” (dyspnoea) and their “intensity of limb discomfort” using Borg’s modified CR10 scale (Borg 1998). The end points were anchored, such that zero represented “no breathing/limb discomfort” and 10 was “the most severe breathing/limb discomfort you have ever experienced or could imagine experiencing”.

### Data capture

Cardiorespiratory and EMG data were averaged over the penultimate 30 s of each exercise bout so as not to conflict with perceptual measures being made in the final 30 s. When calculating LRC, the first 60 s of each 4 min block of exercise were omitted from analysis to allow respiratory patterns (*V*_T_ and *f*-_R_) to stabilise after the static start of arm-cranking; similarly, the last 60 s of each 4 min block of exercise were excluded to account for the recording of perceptual responses and any slowing of cadence that occurred as the participant began to anticipate the rest period. Volume signals from the online system were fed through a signal amplifier (1902, Cambridge Electronic Design, Cambridge, England) and digitised at a sampling rate of 150 Hz using an analogue-to-digital converter (micro 1401 mkII, Cambridge Electronic Design). EMG signals were sampled at 4 kHz, high-pass filtered at 100 Hz, and notch-filtered at 50 Hz to suppress power line and harmonic interference. Data were displayed simultaneously as digital waveforms using integrated data acquisition software (Spike 2 version 7.0, Cambridge Electronic Design). ECG artefact was removed from the EMG waveforms using a custom script procedure similar to that used by others (Alty et al. 2008).

### Statistics

Statistical analysis was performed using SPSS 16.0 for Windows (SPSS Inc., IBM, Chicago, IL, USA). One-way ANOVA with repeated measures was used to test the effect of cadence (50, 70, and 90 rev min^−1^) on cardiorespiratory responses (e.g., $\dot{V}{\text{O}}_{ 2}$, $\dot{V}{\text{CO}}_{ 2}$, $\dot{V}_{\text{E}}$, *V*_T_, *f*_R_, *f*_C_, SpO_2_), perceptual responses, and the prevalence of locomotor-respiratory coupling at each exercise intensity (moderate, heavy). Post-hoc analyses were carried out using Fisher’s LSD. Alpha level was set at 0.05. Data are expressed as mean ± SD unless stated otherwise.

## Results

### Maximal ramp test responses

Peak physiological responses are shown in Table 2. Peak oxygen uptake was variable among participants (24–36 mL kg^−1^ min^−1^), reflecting a wide range of upper-body fitness. Only two participants exhibited a visible plateau in $\dot{V}{\text{O}}_{ 2}$ at end-exercise. Furthermore, the perceived intensity of limb discomfort at exercise cessation was greater than that reported for dyspnoea (10.5 ± 0.5 vs. 7.3 ± 2.0, *p* = 0.021).

**Table 2:** Peak physiological responses to ramp incremental arm-crank exercise

	Rest	Peak
Work rate (W)	–	118 ± 24
$\dot{V}{\text{O}}_{ 2}$ (L min^−1^)	0.30 ± 0.24	2.05 ± 0.41
$\dot{V}{\text{O}}_{ 2}$ (mL kg^−1^ min^−1^)	4.47 ± 0.89	30.3 ± 4.5
$\dot{V}{\text{CO}}_{ 2}$ (L min^−1^)	0.24 ± 0.06	2.64 ± 0.48
RER	0.81 ± 0.07	1.29 ± 0.06
*f*_C_ (b min^−1^)	61 ± 10	169 ± 20
$\dot{V}{\text{O}}_{ 2}$ (L min^−1^)	8.2 ± 1.6	79 ± 17
*V*_T_ (L)	0.59 ± 0.16	2.02 ± 0.51
*f*_R_ (br min^−1^)	14.8 ± 4.3	40.9 ± 8.0
CR10_Limb_	0.0 ± 0.0	10.5 ± 0.5
CR10_Dyspnoea_	0.0 ± 0.0	7.3 ± 2.0

Mean ± SD, *n* = 8

$\dot{V}{\text{O}}_{ 2}$ O_2_ uptake, $\dot{V}{\text{CO}}_{ 2}$ CO_2_ output, *RER* respiratory exchange ratio, *f*_*C*_ cardiac frequency, $\dot{V}{\text{O}}_{ 2}$ minute ventilation, *V*_*T*_ tidal volume, *f*_*R*_ respiratory frequency, *CR10*_*Limb*_ intensity of limb discomfort, *CR10*_*Dyspnoea*_ intensity of breathing discomfort

### Varied-cadence, constant-power test responses

#### Locomotor–respiratory coupling

**Fig. 2:**
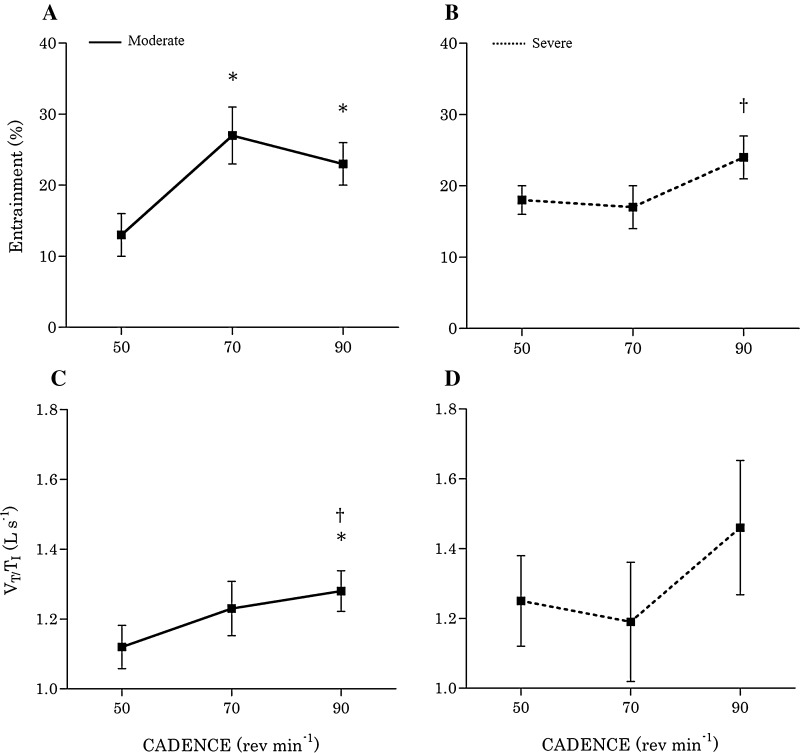
Locomotor–respiratory coupling (entrainment) during moderate (**a**) and severe (**b**) arm-crank exercise, and mean inspiratory flow (*V*_T_/*T*_I_) during moderate (**c**) and severe (**d**) arm-crank exercise performed at 50, 70, and 90 rev min^−1^. During moderate exercise, the prevalence of LRC was highest at 70 rev min^−1^, and during severe exercise at 90 rev min^−1^. Mean inspiratory flow showed similar cadence-mediated patterns. *Significantly different vs. 50 rev min^−1^ (*p* < 0.05); ^†^significantly different vs. 70 rev min^−1^ (*p* < 0.05)

The prevalence of LRC during the varied-cadence, constant-power test is shown in Fig. 2. Participants more frequently demonstrated locomotor-respiratory coupling at high cadences. During moderate exercise, %LRC at 50, 70, and 90 rev min^−1^ was 13 ± 9, 27 ± 10, and 23 ± 8%, respectively, but with no significant differences between 70 and 90 rev min^−1^. During severe exercise, there was no difference in %LRC between 50 and 70 rev min^−1^ (18 ± 5 and 17 ± 8%, respectively), but %LRC was significantly higher at 90 vs. 70 rev min^−1^ (24 ± 7% vs. 17 ± 8%, *p* = 0.034).

#### Cardiorespiratory, diaphragmatic, and perceptual responses

**Fig. 3:**
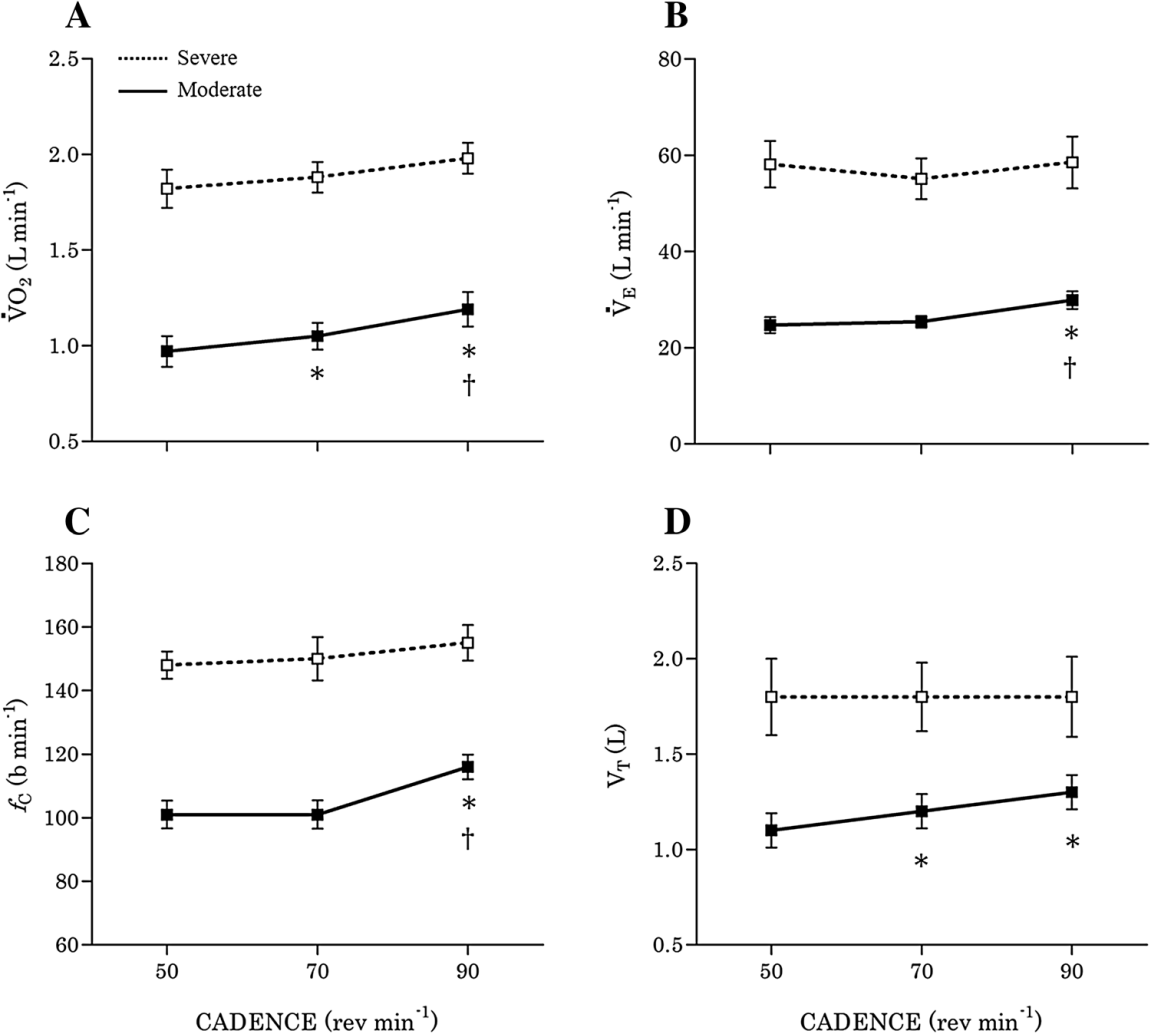
Oxygen uptake (**a**), cardiac frequency (**b**), ventilation (**c**), and tidal volume (**d**) during arm-cranking at moderate and severe intensities, performed at 50, 70, and 90 rev min^−1^. Cardiorespiratory responses during moderate exercise were greater at higher cadences, but the differences were less apparent during severe exercise. *Significantly different vs. 50 rev min^−1^ (*p* < 0.05); ^†^significantly different vs. 70 rev min^−1^ (*p* < 0.05)

Cardiorespiratory responses to the varied-cadence, constant-power exercise test are shown in Table 3. Mean power output during moderate- and severe-intensity exercise was 46 ± 11 W (40 ± 13% *W*_peak_) and 89 ± 12 W (77 ± 18% *W*_peak_), respectively. During moderate exercise, arm-cranking induced greater cardiorespiratory stress at high cadences (Fig. 3). Arm-cranking at 90 rev min^−1^ elicited significantly higher values for $\dot{V}{\text{O}}_{ 2}$ (*p* = 0.001), $\dot{V}{\text{CO}}_{ 2}$ (*p* < 0.001), *f*_C_ (*p* = 0.003), $\dot{V}_{\text{E}}$ (*p* = 0.002), and *V*_T_ (*p* = 0.026) compared to 50 rev min^−1^. Furthermore, $\dot{V}{\text{O}}_{ 2}$ (*p* = 0.018), $\dot{V}{\text{CO}}_{ 2}$ (*p* = 0.006), *f*_C_ (*p* = 0.005), and $\dot{V}_{\text{E}}$ (*p* = 0.013) were higher at 90 compared to 70 rev min^−1^. Concomitantly, dyspnoea during moderate exercise was greatest at 90 rev min^−1^ compared to 70 rev min^−1^ (*p* = 0.021) and 50 rev min^−1^ (*p* = 0.045). Most of the differences in cardiorespiratory function among cadences were attenuated during severe exercise, although there was a trend towards greater $\dot{V}{\text{O}}_{ 2}$ at 90 rev min^−1^ compared to 50 rev min^−1^ (*p* = 0.069). Oxygen saturation (SpO_2_) was unaffected by cadence at either exercise intensity.

**Table 3:** Effects of cadence and exercise intensity on cardiorespiratory, diaphragmatic, and perceptual responses to arm-crank exercise

	Moderate			Severe		
	50 rev min^−1^	70 rev min^−1^	90 rev min^−1^	50 rev min^−1^	70 rev min^−1^	90 rev min^−1^
$\dot{V}{\text{O}}_{ 2}$ (L min^−1^)	0.97 ± 0.24	1.05 ± 0.21*	1.19 ± 0.25*^†^	1.82 ± 0.27	1.88 ± 0.23	1.98 ± 0.24
$\dot{V}{\text{O}}_{ 2}$ (mL kg^−1^ min^−1^)	14.3 ± 2.9	15.5 ± 2.5*	17.5 ± 2.8*^†^	27.0 ± 2.7	27.9 ± 1.8	29.4 ± 1.4
$\dot{V}{\text{CO}}_{ 2}$ (L min^−1^)	0.94 ± 0.24	1.03 ± 0.24*	1.20 ± 0.26*^†^	1.96 ± 0.31	1.93 ± 0.26	2.03 ± 0.34
RER	0.97 ± 0.11	0.97 ± 0.10	1.00 ± 0.06	1.08 ± 0.09	1.03 ± 0.10	1.02 ± 0.09
$\dot{V}_{\text{E}} /\dot{V}{\text{O}}_{ 2}$	26.1 ± 4.0	24.8 ± 4.2	25.5 ± 3.1	31.9 ± 5.9	29.5 ± 6.3	29.3 ± 5.5
$\dot{V}_{\text{E}} /\dot{V}{\text{CO}}_{ 2}$	27.2 ± 5.2	25.8 ± 5.6	25.6 ± 3.6	29.5 ± 3.9	28.6 ± 4.9	28.7 ± 4.0
*f*_C_ (b min^−1^)	101 ± 12	101 ± 13	116 ± 11*^†^	148 ± 12	150 ± 19	155 ± 16
$\dot{V}_{\text{E}}$ (L min^−1^)	24.7 ± 4.7	25.4 ± 3.4	29.9 ± 5.2*^†^	58.1 ± 13.7	55.1 ± 12.0	58.5 ± 15.2
*V*_T_ (L)	1.12 ± 0.24	1.23 ± 0.24*	1.28 ± 0.25*	1.82 ± 0.57	1.76 ± 0.51	1.78 ± 0.58
*f*_R_ (br min^−1^)	22.8 ± 4.9	21.3 ± 4.8	24.3 ± 6.5	33.3 ± 7.4	34.0 ± 13.7	35.8 ± 13.2
*T*_I_ (s)	1.16 ± 0.18	1.25 ± 0.29	1.02 ± 0.17*^†^	1.52 ± 0.50	1.61 ± 0.65	1.33 ± 0.60^†^
*T*_E_ (s)	1.41 ± 0.28	1.36 ± 0.20	1.39 ± 0.43	0.91 ± 0.26	0.97 ± 0.27	0.84 ± 0.23^†^
*T*_TOT_ (s)	2.57 ± 0.44	2.61 ± 0.45	2.40 ± 0.55	2.43 ± 0.62	2.58 ± 0.85	2.17 ± 0.74^†^
*T*_I_/*T*_TOT_	0.45 ± 0.03	0.47 ± 0.04	0.43 ± 0.05	0.62 ± 0.10	0.61 ± 0.08	0.60 ± 0.10
*T*_E_/*T*_TOT_	0.55 ± 0.03	0.53 ± 0.04	0.57 ± 0.05	0.38 ± 0.10	0.39 ± 0.08	0.40 ± 0.10
*V*_T_/*T*_I_ (L s^−1^)	1.12 ± 0.15	1.23 ± 0.21	1.28 ± 0.16*^†^	1.25 ± 0.36	1.19 ± 0.40	1.46 ± 0.54
SpO_2_ (%)	100 ± 0	100 ± 0	100 ± 0	100 ± 0	100 ± 0	100 ± 1
EMG_di,RMS_ (%max)	34 ± 23	22 ± 7	40 ± 20	89 ± 18	88 ± 17	83 ± 19
CR10_Limbs_	2.3 ± 1.0	2.8 ± 1.1	3.3 ± 1.2	8.3 ± 2.2	7.5 ± 1.7	7.0 ± 2.2
CR10_Dyspnoea_	1.9 ± 1.7	1.7 ± 1.4	2.6 ± 1.4*^†^	5.7 ± 3.1	5.1 ± 2.9	5.4 ± 3.1

Mean ± SD, *n* = 8

$\dot{V}{\text{O}}_{ 2}$ O_2_ uptake, $\dot{V}{\text{CO}}_{ 2}$ CO_2_ output, *RER* respiratory exchange ratio, $\dot{V}_{\text{E}} /\dot{V}{\text{O}}_{ 2}$ ventilatory equivalent for O_2_, $\dot{V}_{\text{E}} /\dot{V}{\text{CO}}_{ 2}$ ventilatory equivalent for CO_2_, *f*_*C*_ cardiac frequency, $\dot{V}_{\text{E}}$ minute ventilation, *V*_*T*_ tidal volume, *f*_*R*_ respiratory frequency, *T*_*I*_ inspiratory time, *T*_*E*_ expiratory time, *T*_*TOT*_ total respiratory time, *T*_*I*_ */T*_*TOT*_ inspiratory duty cycle, *T*_*E*_ */T*_*TOT*_ expiratory duty cycle, *V*_*T*_ */T*_*I*_ mean inspiratory flow, *SpO*_*2*_ arterial oxygen saturation, *EMG*_*di,RMS*_ electromyographic activity (root mean square) of the diaphragm, *CR10-*_*Limbs*_ intensity of limb discomfort, *CR10*_*Dyspnoea*_ intensity of breathing discomfort

* Significantly different from 50 rev min^−1^ (*p* < 0.05); ^†^ significantly different from 70 rev min^−1^ (*p* < 0.05)

During moderate exercise, inspiratory time (*T*_I_) was shorter at 90 rev min^−1^ compared to 50 rev min^−1^ (*p* = 0.012) and 70 rev min^−1^ (*p* = 0.016), and during severe exercise, *T*_I_ was shorter at 90 rev min^−1^ compared to 70 rev min^−1^ (*p* = 0.049). Mean inspiratory flow (*V*_T_/*T*_I_) during moderate exercise was also greater at 90 rev min^−1^ compared to both 70 rev min^−1^ (*p* = 0.009) and 50 rev min^−1^ (*p* = 0.001). We were able to obtain phasic diaphragm EMG traces from four participants. In these participants, diaphragm activity was substantially greater during severe vs. moderate exercise (87 ± 18 vs. 32 ± 17% EMG_di_ max), but with no discernible differences among cadences at either intensity.

## Discussion

The aim of this study was to assess the acute influence of arm-crank cadence and work rate on locomotor-respiratory coupling and cardiorespiratory function. A novel finding was that participants more frequently synchronised their locomotor and respiratory rhythms when arm-cranking at moderate-high cadences (70–90 rev min^−1^), compared to low cadences (50 rev min^−1^). Furthermore, arm-cranking at 90 rev min^−1^ significantly increased cardiorespiratory stress during moderate exercise when compared to either 50 or 70 rev min^−1^, with correspondingly greater perceived intensity of dyspnoea. Collectively, these findings suggest that, for a given submaximal work rate, there may be a strong cadence-mediated influence on cardiorespiratory function, dyspnoea, and respiratory entrainment patterns, as per our original hypothesis.

Participants exhibited LRC more often at higher arm-crank cadences (70–90 rev min^−1^) during both moderate and severe exercise. It has been suggested that participants engage in LRC to simplify the coordination of respiratory and postural tasks, particularly during dynamic exercise when loads on the torso exacerbate the mechanical demands of the respiratory muscles (Hodges et al. 2001). The greater prevalence of LRC, therefore, is likely associated with elevated thoracic postural demands imposed by the cadence rate.

Our observations reflect that of the previous research which has also observed LRC to occur with a prevalence of ~25% during arm-cranking performed at 90 rev min^−1^, as well as a magnitude of LRC (~25%) that appears to be independent of work rate (Paterson et al. 1986). Early studies of LRC that used cross correlation to detect relationships between trains of respiratory and locomotor impulses also observed an increased tendency to entrain when exercising at faster cadences, although such measurements were made during lower-limb cycle ergometry (Bechbache and Duffin 1977).

Phase-locking of locomotor and respiratory patterns reduces the mechanical interactions between locomotion and ventilation, and might, therefore, minimise the conflict between muscles that contribute to both (Deban and Carrier 2002). As such, LRC occurs most frequently during periods of heightened respiratory muscle conflict to reduce the energy cost of breathing (Daley et al. 2013). Since the primary mechanical factors controlling LRC are thoracic loading and inertial displacement of soft body tissues (Bramble and Carrier 1983; Bramble and Jenkins 1993), it is likely that the fast, rhythmical rotations of the arms and shoulders during arm-cranking exacerbated the mechanical interactions between locomotor and ventilatory muscular contractions. The present observation that LRC is more prevalent at 90 rev min^−1^ during severe exercise is indirect evidence that such conditions result in additional mechanical demands on the thoracic complex.

The present findings suggest a causal relationship between the greater prevalence of LRC and the higher cadence rate. We found that moderate exercise at 90 rev min^−1^ resulted in greater mean inspiratory flow compared to either 70 or 50 rev min^−1^ (1.28 ± 0.16 vs. 1.23 ± 0.21 vs. 1.12 ± 0.15 L s^−1^). Similar patterns were noted for *T*_I_ and *T*_TOT_ at both intensities, in that there were shorter respiratory duty cycles at 90 rev min^−1^, indicative of faster inspiration to expiration transitions. Others have found that when engaged in LRC, healthy participants tend to initiate the inspiration to expiration transition at mechanically compatible (assistive) phases of the locomotor cycle to facilitate, rather than impede, pulmonary airflow; resulting in faster inspiration to expiration transitions (Daley et al. 2013). Since we observed no differences in respiratory frequency across cadences at either work rate, it appears that our participants entrained more frequently at higher cadences to facilitate lung expansion via significant increases in expiratory and inspiratory flow. This is in contrast with earlier suggestions that greater LRC at faster cadences may result in greater respiratory frequency (Price et al. 2007). Collectively, these observations are consistent with the hypothesis that LRC results from heightened respiratory muscle conflict to facilitate respiratory flow. Since isometric contractions elicit the same $\dot{V}{\text{O}}_{ 2}$ per unit of muscle mass as dynamic muscle contractions (Elder et al. 2006), faster cadence arm ergometry may have resulted in postural muscle contractions that elevated $\dot{V}{\text{O}}_{ 2}$ without directly contributing to force output or propulsion of the crank shaft. Such responses have been reported to reduce gross and net mechanical efficiency (Powers et al. 1984).

In an effort to quantify the combined ventilatory and reactive postural contractions of the respiratory muscles, we measured neural drive to the diaphragm during exercise. Since the diaphragm has both inspiratory and static postural functions (Hodges et al. 1997; Hodges and Gandevia 2000), it was expected that greater postural disturbances at high cadences would manifest in greater neural drive to the diaphragm, and yet, the present data do not support this hypothesis. Although diaphragm EMG may increase during static postural contractions (Hodges et al. 1997; Hodges and Gandevia 2000), isometric rotational tasks do not elicit significant increases in diaphragm EMG (Hudson et al. 2010). Furthermore, when ventilation increases during dynamic exercise, neural input to the diaphragm is altered to prioritise its ventilatory functions (Hodges et al. 2001), resulting in diminished postural drive. Although we were currently unable to assess phrenic postural input, it appears that the role of the diaphragm in postural support at high cadences may be minimal, as suggested by the present data. Since inspiratory drive is distributed differently across various inspiratory muscles, possibly according to their mechanical effectiveness (Butler 2007), it may be that other trunk muscles—those with a less dominant role in pulmonary ventilation—make a more functional contribution to isometric postural support during high-cadence arm-cranking. Further assessment of the accessory respiratory muscles, deep muscles of the abdomen, and superficial muscles of the lower back may yield additional insight into their respective postural contributions during dynamic upper-body exercise.

The influence of cadence on cardiorespiratory function was reduced during severe exercise, as also noted in the previous studies on arm-cranking (Powers et al. 1984). This attenuated response at high intensities may relate to the greater work rate of severe compared to moderate exercise (77 ± 18 vs. 40 ± 13% *W*_peak_). It has been suggested that unloaded arm-cranking at high cadences elicits a greater energy cost of moving the exercising limbs compared to low cadences (Price et al. 2007). As the power output (and subsequent energy demand) increases, however, the energy cost associated with unloaded arm-cranking becomes an increasingly smaller contributor to total energy expenditure. Moreover, generating a high absolute external power output during severe exercise likely requires a greater volume of active muscle mass, at both high and low cadences, to revolve the flywheel. Cardiorespiratory function, therefore, is ultimately dictated by the greater volume of muscle mass recruited during high-intensity arm-cranking, thus minimising the cadence influence on $\dot{V}{\text{O}}_{ 2}$ at this work rate.

### Implications

Our findings may have practical implications for both patients and healthy subjects. Since all primary and secondary muscles of respiration attach to the ribs, the accessory (non-respiratory) muscular contractions associated with upper-body exercise, might produce substantial distortion and stiffening of the ribcage (Kenyon et al. 1997). This, in turn, likely inhibits the expansion of tidal volume during upper-body exercise, thereby increasing sensations of breathlessness in patients with obstructive respiratory disorders. Upper-body exercise is typically incorporated into rehabilitation programmes, so we recommend that the exercise is performed at low cadences which are likely to offset dyspnoea intensity. Furthermore, additional loading of the expiratory muscles during high-intensity (high-cadence) upper-body exercise might contribute to expiratory muscle fatigue following arm-cranking (Tiller et al. 2016), which may impede exercise capacity via the respiratory muscle metaboreflex response (Dempsey et al. 2006) during which there is an increased competition for cardiac output. Respiratory muscle fatigue has also been shown to increase the intensity of dyspnoea (Suzuki et al. 1992) and, therefore, athletic populations should also carefully consider the cadence rate applied during upper-limb exercise.

In conclusion, the greater prevalence of LRC during moderate–high-cadence arm-cranking, and the associated facilitation of respiratory airflow, supports the hypothesis that such exercises result in greater reactive postural contractions. Increased isometric work might also contribute to the greater O_2_ cost of high-cadence arm-cranking. Since reactive muscle contractions do not contribute directly to locomotion or force development, activities that impose large reactive forces appear to impact negatively on oxygen economy and dyspnoea.

## Abbreviations

UBE: Upper-body exercise
LRC: Locomotor–respiratory coupling
GET: Gas-exchange threshold
EMG: Electromyography
ECG: Electrocardiography
RMS: Root mean square
SD: Standard deviation
ANOVA: Analysis of variance
